# Overall readmissions and readmissions related to dehydration after creation of an ileostomy: a systematic review and meta-analysis

**DOI:** 10.1007/s10151-022-02580-6

**Published:** 2022-02-22

**Authors:** I. Vogel, M. Shinkwin, S. L. van der Storm, J. Torkington, J. A.Cornish, P. J. Tanis, R. Hompes, W. A. Bemelman

**Affiliations:** 1 Department of Surgery, G4, University of Amsterdam, P.O. Box 22660, 1100DD Amsterdam, The Netherlands; 2 Department of Colorectal Surgery, Cardiff & Vale University Health Board, Cardiff, UK

**Keywords:** Ileostomy, Readmission, Dehydration, High output stoma

## Abstract

**Background:**

Hospital readmissions after creation of an ileostomy are common and come with a high clinical and financial burden. The aim of this review with pooled analysis was to determine the incidence of dehydration-related and all-cause readmissions after formation of an ileostomy, and the associated costs.

**Methods:**

A systematic literature search was conducted for studies reporting on dehydration-related and overall readmission rates after formation of a loop or end ileostomy between January 1990 and April 2021. Analyses were performed using R Statistical Software Version 3.6.1.

**Results:**

The search yielded 71 studies (*n* = 82,451 patients). The pooled incidence of readmissions due to dehydration was 6% (95% CI 0.04–0.09) within 30 days, with an all-cause readmission rate of 20% (CI 95% 0.18–0.23). Duration of readmissions for dehydration ranged from 2.5 to 9 days. Average costs of dehydration-related readmission were between $2750 and $5924 per patient. Other indications for readmission within 30 days were specified in 15 studies, with a pooled incidence of 5% (95% CI 0.02–0.14) for dehydration, 4% (95% CI 0.02–0.08) for stoma outlet problems, and 4% (95% CI 0.02–0.09) for infections.

**Conclusions:**

One in five patients are readmitted with a stoma-related complication within 30 days of creation of an ileostomy. Dehydration is the leading cause for these readmissions, occurring in 6% of all patients within 30 days. This comes with high health care cost for a potentially avoidable cause. Better monitoring, patient awareness and preventive measures are required.

**Supplementary Information:**

The online version contains supplementary material available at 10.1007/s10151-022-02580-6.

## Introduction

Hospital readmissions after creation of an ileostomy are common and impede patient convalescence [1]. Reasons for readmission after fecal diversion include stoma-related problems, such as dehydration, stoma outlet obstruction, peristomal skin problems, anastomotic leak, and generic post-operative complications (e.g., infection or thrombo-embolic events).

Dehydration is often cited as a leading cause for stoma-related readmissions, due to fluid and electrolyte losses [2]. Dehydration can contribute to substantial post-operative morbidity, increasing the risk of acute renal failure, electrolyte derangement, and even cardiac arrhythmias [3]. There is a growing consensus that these readmissions place a significant burden on patients and are costly for the healthcare system, but that they might also be avoidable to some extent [4–6].

The reported incidence of readmission particularly in relation to dehydration varies [6–8], probably due to inconsistent definitions, and completeness and duration of post-operative follow-up. To quantify the risks and benefits of an ileostomy, to reduce stoma-related readmissions, and to guarantee patient safety, the scope of the problem needs to be clear. Therefore, the aim of this systematic review was to assess the prevalence of readmission related to dehydration after the creation of an ileostomy. The secondary aims included overall readmissions and their causes after creation of an ileostomy as well as cost implications.

## Materials and methods

This review was conducted in line with the Cochrane Handbook for systematic reviews of In Reporting following the Preferred Reporting Items for Systematic Reviews and Meta-Analyses (PRISMA) and the Meta-analysis Of Observational Studies in Epidemiology (MOOSE) guidelines [9]. The study protocol was registered in PROSPERO, the international prospective register of systematic reviews (registration number CRD42021231472). Comprehensive literature searches were conducted using PubMed, Embase, and Cochrane databases for articles published from January 1990 until April 2021. The full search strategy is displayed in Supplementary Table S1–3.

Studies were considered for inclusion if they met the following criteria: (1) patients with a newly created loop or end ileostomy for any indication; (2) assessment of readmissions related to dehydration, or overall number of readmissions, or other reasons for readmission after creation of an ileostomy; (3) studies were cohort, case-matched studies, or randomized clinical trials. The exclusion criteria were: (1) reviews, letters, expert opinions, commentaries, case reports, or case series with less than 10 cases; (2) language other than English; (3) lack of the sufficient data or outcomes of interest; (4) visits just to the emergency department; (5) studies reporting only on complications of revised ileostomies (with exception of readmissions for a revision of a newly created ileostomy); (6) second stage ileostomies in a three-stage ileo-anal pouch procedure; (7) colostomies, jejunostomies, non-intestinal stomas, and ghost ileostomies; (8) duplicate studies.

Two reviewers (IV and MS) independently reviewed titles and abstracts, followed by full-text revision. Disagreements were resolved by consensus discussion between the two reviewers (IV and MS).

### Data extraction and quality assessment

Data were extracted independently by two authors (IV and MS) and included the following variables: year of publication, country, study design, number of patients, characteristics of included patients, indication for the ileostomy, type of surgery, number of elective procedures, number of open procedures, type of stoma (loop/end), overall number of readmissions, number of readmissions related to dehydration, other reasons for readmissions, duration, and cost of readmissions related to dehydration.

The indications for an ileostomy were recorded and were classified as colorectal disease if they included bowel cancer, inflammatory bowel disease, diverticulitis, or familiar adenomatous polyposis.

Readmissions were defined as an unplanned return to the hospital with an overnight stay for any reason. This did not include elective or planned readmissions.

The following were accepted as readmission related to dehydration: a clinician-reported diagnosis of dehydration, or high output stoma (defined as ≥ 1500 mL stoma production in 24 h, or the Kidney Disease Global Improving guideline definition of acute kidney injury which includes any of the following: absolute increase in serum creatinine ≥ 0.3 mg/dL in a 48-h period, 1.5-fold increase in serum creatinine level in a 48-h period, or oliguria of ≤ 0.5 mL/kg for ≥ 6 h [10, 11].

Readmissions for infection included all pathology (such as chest infections and urinary tract infections). It did not include anastomotic leaks, which were reported separately.

Whilst the primary outcome was readmission within 30 days related to dehydration after creation of an ileostomy readmission for other timeframes was also summarised. Secondary outcomes included number of all-cause readmissions, other common indications for readmission, duration, and cost associated with readmission.

All included studies were assessed for methodological quality and risk of bias. For cohort studies, the Newcastle Ottawa quality assessment scale was used to assess risk of bias [12]. For randomized controlled trials, the Jadad scoring system was used [13]. When the randomized controlled trials (RCTs) groups were not analysed as described in the RCT, the Newcastle Ottawa quality assessment was used. Two of the authors (IV and MS) performed the quality assessment, with discussion of conflicts to achieve consensus.

### Statistical analysis

Quantitative analysis was performed using RStudio (R Software version 3.6.1-©2009–2012, RStudio, Inc. software) with a random-effects model. For the outcome measures, pooled weighted proportions with corresponding 95% CIs were calculated using inversed variance weighting. Heterogeneity was assessed using the *I*^2^ and *τ*^2^ statistics, and the data were considered significant if the *p* value (*τ*^2^) was < 0.1 with low, moderate, and high for *I*^2^ values of 25%, 50%, and 75%.

## Results

**Fig. 1:**
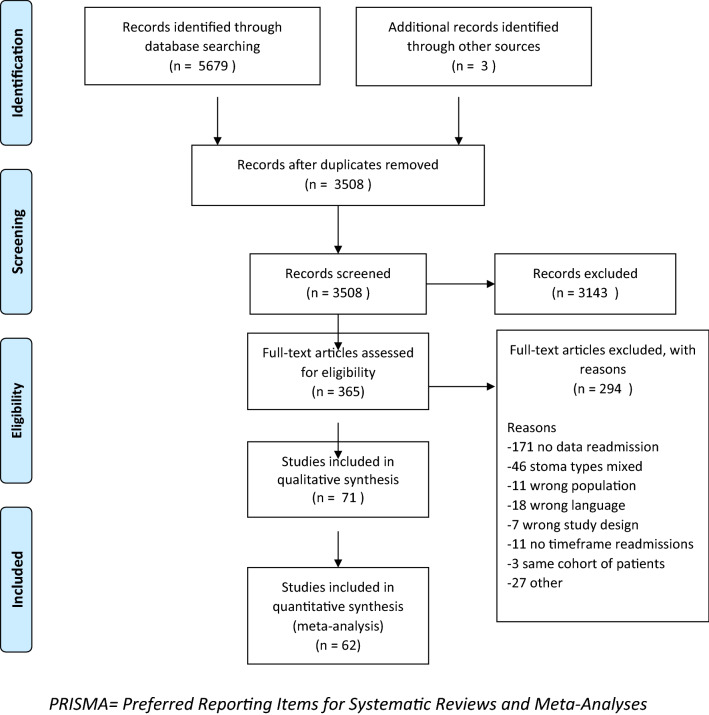
PRISMA flow diagram

In total, 3508 articles were screened on title and abstract, with 3143 articles not meeting our inclusion criteria. A further 294 studies were excluded after full-text review leaving 71 studies (82,451 patients) for analysis, with 62 studies able to be included in a quantitative meta-analysis (Fig. 1). The assessment for methodological quality and risk of bias is described in Table 1.

**Table 1:** Assessment for methodological quality and risk of bias

Author	Country	Jadad score	Newcastle quality	Ottawa assessment		
		Total	Selection (0–4)	Comparability (0–2)	Outcome (0–3)	Total (0–9)
Van Loon 2020	US		***	*	**	6
Lee 2020	Korea		***	*	**	6
Liu 2020	New Zealand		***	*	**	6
Kim 2020	US		***	*	***	7
Yaegasgi 2019	Japan		***	**	**	7
Hendren 2019	USA		**	*	**	5
Schineis 2019	Germany		***	*	**	6
Grahn 2019	US	6.5				
Fielding 2019	UK		**	*	**	5
Alqahtani 2019	USA		***	*	**	6
Karjalainen 2019	Finland		***	*	**	6
Lee J 2019	Mexico		***	**	**	7
Gonella 2019	Italy		***	**	**	7
Chen 2018	USA		***	**	***	8
Justinianio 2018	USA		***	**	***	8
Sier 2018	The Netherlands	6.5				
Charak 2018	US		***	*	**	6
Kandagatla 2018	US		***	**	**	7
Bednarski 2018	US		****	*	***	8
Park 2018	Sweden		***	*	**	6
Migdanis 2018	Greece	6.5				
Iqbal 2018	US		***	*	**	6
Wen 2017	US		***	*	**	6
Shaffer 2017	US		**		**	4
Yin 2017	Taiwan		***	**	**	7
Li L 2017	US		**	*	**	5
Fish 2017	US		**	*	**	5
Iqbal 2017	US		***	*	**	6
Shwaartz 2017	US		***	*	**	6
LI W 2017	US		****	*	**	6
Shah 2017	US		***	*	**	6
Hawkins 2016	US		****	*	**	7
Tseng 2016	US		****	**	**	8
Helavirta 2016	Finland		***	*	**	6
Anderin 2016	Sweden		***	*	**	6
Kulaylat 2015	US		**	*	**	5
Pellino 2014	Italy		***	*	**	6
Hardiman 2014	US		***		**	5
Tyler 2014	US		**	*	**	5
Phatak 2014	US		***	*	**	6
Abegg 2014	The Netherlands		***	*	***	7
Glasgow 2014	US		***	*	***	7
Feroci 2013	Italy		***	*	**	6
Parnaby 2013	UK		**	**	**	6
Coakley 2013	US		***	*	**	6
Gu 2013	US		***		**	5
Hardt 2013	Germany		***	*	**	6
Byrne 2013	UK		**		**	4
Paquette 2013	South Korea	6.5				
Lee S 2013	South Korea	6				
Jafari 2013	US		***	*	**	6
Akesson 2012	Sweden		***	*	***	7
Duff 2012	Australia		****		**	5
Nagle 2012	US		***	*	**	6
Marsden 2012	UK		****	*	**	7
Messaris 2012	US		***	**	**	7
Chun 2012	US		***	**	**	7
Gessler 2012	Sweden		***	**	***	8
Beck 2011	Germany		***	*	**	6
Fajardo 2010	US		***	**	**	7
Telem 2010	US		***	*	**	6
Datta 2009	Canada		***	**	***	8
Kariv 2007	US		***	*	**	6
Fowkes 2008	UK		***		**	5
Schwenk 2006	Germany		**		**	4
Larson 2006	US		***	**	**	7
Garcia-Botello 2004	Spain		****	**	**	8
Hallbook 2002	Sweden		***	*	***	7
Okamoto 1995	Japan		***	*	*	5
Wexner 1993	US		****	*	*	6
Winslet 1991	UK		***	*	**	6

*represents one point

### Study characteristics

Baseline characteristics of the studies are summarised in Table 2. All patients received a newly created loop or end ileostomy. Indications for an ileostomy varied widely from colorectal cancer, inflammatory bowel disease, diverticulitis, familiar adenomatous polyposis, and gynecological malignancies, to any other indication for an ileostomy. Elective/emergency intention was reported in 42 studies, with the majority of patients included (76.9%) undergoing elective surgery [1–4, 7, 11, 14–49]. Thirty-six studies reported method of access; in 41.9% stoma creation was carried out with an open approach [3, 8, 11, 14–17, 19, 20, 22, 25, 28, 31, 32, 34–39, 42, 43, 45, 46, 49–59].

**Table 2:** Patient and study characteristics

Author	Design	Patients *N*	Female *N* (%)	Age years	ASA > 3 *N* (%)	Underlying disease	Type of surgery	Elective *N* (%)	Open *N* (%)	Stoma type	Readmission overall *N* (%)	Readmission dehydration *N* (%)	Time frame readmissions
Van Loon 2020	Retrospective	393	195 (50)	–	–	Colorectal disease	Colorectal resection	–	–	Both	117 (30)	34 (9)	30 days
Lee N 2020	Retrospective	302	99 (33)	–	14 (5)	Rectal cancer	LAR	–	5 (2)	Loop	51 (17)	20 (7)	6 months
Liu 2020	Retrospective	266	141 (53)	–	108 (41)	Any	Any	159 (60)	118 (44)	Both	78 (29)	23 (9)	60 days
Kim 2020	Retrospective	39,380	19,375 (49)	–	6531 (17)	Colorectal disease	Any	30,593 (78)	7824 (20)	Both	5718 (15)	227 (0.6)	30 days
Yaegasgi 2019	Case-matched	58	17 (29)	60 (IQR 50–66)	–	Rectal cancer	LAR	–	–	Loop	–	6 (11)	Creation and closure
Hendren 2019	Retrospective	982	488 (50)	–	–	Colorectal disease	Any	500 (51)	665 (68)	Both	200 (20)	–	30 days
Schineis 2019	Retrospective	180	76 (42)	41 (R 18–86)	–	UC	Colectomy	149 (83)	15 (8)	End	14 (8)	–	30 days
Grahn 2019	RCT	100	55 (55)	–	74 (74)	Any	Any	88 (88)	–	Both	20 (20)	7 (7)	30 days
Fielding 2019	Retrospective	426	187 (44)	68 (IQR 61–74)	74 (17)	Rectal cancer	Rectal resection	426 (100)	–	Loop	134 (32)	–	1 year
Alqahtani 2019	Retrospective	15,222	7272 (48)	61 (IQR 44–72)	936 (6)	Colorectal disease	Any	11,531 (58)	11,841 (22)	Loop	–	315 (2)	30 days
Karjalainen 2019	Retrospective	119	28 (24)	43 (SD 13)	–	UC	Procto–colectomy	–	119 (100)	Loop	50 (42)	19 (16)	3 months
Lee J 2019	Retrospective	208	105 (51)	59 (IQR 49–70)	137 (66)	Diverticulitis	Colectomy	0	172 (83)	Loop	23 (11)	–	30 days
Gonella 2019	Retrospective	296	116 (39)	–	–	Any	Any	185 (63)	–	–	53 (18)	20 (7)	30 days
Chen 2018	Retrospective	8064	3646 (45)	55 (IQR 43–65)	3965 (49)	Colorectal disease	Any	7538 (91)	5143 (64)	Both	1620 (20)	234 (3)	30 days
Justinianio 2018	Retrospective	262	123 (47)	54	–	Colorectal disease	Colorectal resection	174 (66)	115 (44)	Both	78 (30)	29 (11)	30 days
Sier 2018	RCT	339	130 (38)	60 (SD 14)	29 (9)	Colorectal disease	Any	339 (100)	–	Both	21 (6)	–0	30 days
Charak 2018	Retrospective	99	48 (48)	52 (SD 19)	55 (56)	Colorectal disease	Colorectal resection	99 (100)	43 (43)	Loop	36 (36)	14 (14)	60 days
Kandagatla 2018	Retrospective	360	170 (47)	48	206 (58)	Colorectal disease	Any	223 (62)	–	Both	98 (27)	15 (4)	30 days
Bednarski 2018	Retrospective	49	19 (39)	51 (R 22–75)	–	Colorectal cancer	Colorectal resection	–	–	Loop	15 (31)	4 (8)	60 days
Park 2018	Retrospective	71	24 (34)	39 (R 16–21)	3 (4)	UC	Procto-colectomy	71 (100)	–	Loop	13 (18)	8 (11)	90 days
Migdanis 2018	RCT	80	26 (32)	66 (SD 12)		Colorectal disease	LAR	80 (100)	–	Loop	15 (19)	10 (13)	30 days
Iqbal 2018	Retrospective	86	43 (50)	54	71 (82)	Colorectal disease	LAR	86 (100)	33 (38)	Loop	22 (26)	8 (9)	30 days
Wen 2017	Case–matched	74	–	–	–	Colorectal disease	Colorectal resection	74 (100)	–	Both	12 (16)	3 (4)	30 days
Shaffer 2017	Retrospective	162	–	–	–	Any	Colorectal resection	–	–	–	29 (18)	–	30 days
Yin 2017	Retrospective	28	9 (32)	64 (SD 12)	–	Rectal cancer	LAR	27 (96)	–	Loop	10 (36)	–	Creation and closure
Li L 2017	Retrospective	84	1 (1)	–	–	Colorectal cancer	Colorectal resection	–	58 (69)	Both	–	14 (17)	1 year
Fish 2017	Retrospective	407	183 (45)	53 (SD 16)	–	Colorectal disease	Any	317 (78)	220 (54)	Both	113 (28)	47 (12)	60 days
Iqbal 2017	Prospective	55	–	55	–	Colorectal disease	Colorectal resection	–	–	Both	–	20 (36)	30 days
Shwaartz 2017	Retrospective	204	100 (49)	62 (SD 15)	141 (69)	Colorectal disease	Any	150 (74)	164 (80)	Both	31 (15)	–	30 days
LI W 2017	Retrospective	1267	547 (43)	47	586 (46)	Colorectal disease	Colorectal resection	1236 (98)	1021 (81)	Loop	163 (13)	38 (3)	30 days
Shah 2017	Retrospective	192	–	–	–	Colorectal disease	Colorectal resection	192 (100)	–	Both	39 (20)	–	30 days
Hawkins 2016	Prospective	186	113 (60)	57 (SD20)	136 (73)	Colorectal disease	Ileocecal resection	133 (72)	70 (38)	Loop	42 (23)	–	30 days
Tseng 2016	Retrospective	44	–	63 (R 54–91)	–	Ovarian cancer	Any	–	–	Loop	10 (23)	2 (5)	30 days
Helavirta 2016	Retrospective	133	–	–	–	UC	Procto-colectomy	–	–	Loop	–	9 (7)	30 days
Anderin 2016	Retrospective	139	52 (37)	62 (R 30–84)	13 (9)	Rectal cancer	LAR	–	–	Loop	22 (16)	5 (4)	3 years
Kulaylat 2015	Retrospective	381	–	–	–	Colorectal disease	Any	–	10 (100)	Both	154 (40)	–	30 days
Pellino 2014	Prospective	10		88 (R 84–90)	–	UC	Procto-colectomy	–	–	Loop	–	1 (10)	2 weeks
Hardiman 2014	Retrospective	430	222 (52)	50	–	Any	Any	255 (59)	–	Both	110 (26)	–	30 days
Tyler 2014	Retrospective	6007	2894 (48)	60 (SD 17)	–	Colorectal disease	Any	3046 (51)	–	Both	1484 (25)	–	30 days
Phatak 2014	Retrospective	294	95 (32)	56 (SD 13)		Rectal cancer	Rectal resection	294 (100)	264 (89)	Loop	63 (21)	32 (11)	60 days
Abegg 2014	Retrospective	118	41 (36)	65 (IQR 60–72)	6 (5)	Colorectal cancer	Colorectal resection	–	–	Loop	31 (26)	–	Creation and closure
Glasgow 2014	Retrospective	53	53 (100)	63 (SD 11)	–	Gynecologic malignancy	Any	–	–	Both	18 (34)	13 (25)	30 days
Feroci 2013	Prospective	59	–	–	–	Colorectal disease	Any	59 (100)	–	Loop	0	0	30 days
Parnaby 2013	Case-matched	64	38 (59)	41 (R 24–55)	8 (13)	UC	Subtotal colectomy	20 (31)	32 (50)	Loop	12 (19)	–	30 days
Coakley 2013	Retrospective	107	41 (38)	38 (SD 17)	47 (44)	UC	Colectomy	–	82 (77)	Loop	14 (13)	–	30 days
Gu 2013	Retrospective	204	99 (49)	35 (R 18–75)	–	UC	Total colectomy	–	9 (4)	End	35 (17)	4 (2)	30 days
Hardt 2013	Retrospective	103	36 (35)	62	26 (25)	Rectal cancer	Rectal resection	103 (100)	70 (68)	Loop	2 (2)	00	14 days
Byrne 2013	Prospective	20	8 (40)	64 (R 41–84)	1 (5)	Rectal cancer	LAR	20 (100)	2 (10)	Loop	2 (10)	–	30 days
Paquette 2013	Retrospective	201	92 (46)	47 (SD 17)	–	Colorectal disease	Any	–	191 (95)	Both	–	33 (17)	30 days
Lee S 2013	RCT	98	34 (35)	61	9 (9)	Rectal cancer	LAR	98 (100)	0	Loop	0	00	30 days
Jafari 2013	Retrospective	991	629 (64)	60 (SD 12)	427 (43)	Rectal cancer	LAR	–	–	Loop	201 (20)	–	30 days
Akesson 2012	Retrospective	92	38 (41)	66 (SD 2)	13 (14)	Colorectal disease	LAR	–	–	Loop	29 (32)	13 (14)	30 days
Duff 2012	Prospective	75	41 (55)	35 (R 15–72)	–	UC	Procto-colectomy	–	0	Loop	18 (24)	6 (8)	30 days
Nagle 2012	Prospective	203	101 (50)	51	–	Colorectal disease	Any	–	–	Both	66 (32)	25 (12)	30 days
Marsden 2012	Prospective	54	16 (30)	71	11 (20)	Rectal cancer	LAR	54 (100)	2 (4)	Loop	12 (22)	–	30 days
Messaris 2012	Retrospective	603	268 (44)	48 (SD 18)	77 (13)	Colorectal disease	Any	509 (84)	540 (90)	Loop	102 (17)	44 (7)	60 days
Chun 2012	Retrospective	123	54 (44)	49 (R 12–69)	–	Colorectal disease	Any	123 (100)	–	Loop	–	14 (11)	Creation and closure
Gessler 2012	Retrospective	262	88 (34)	67 (R 23–95)	–	Colorectal cancer	Any	224 (85)	–	Loop	41 (16)	20 ( 8)	30 days
Beck 2011	Retrospective	107	45 (42)	63 (R 21–90)	–	Any indication	Any	–	–	Loop	–	6 (6)	Creation and closure
Fajardo 2010	Retrospective	124	63 (51)	40 (R 15–78)	–	UC or FAP	IPPA	124 (100)	69 (56)	Loop	–	13 (10)	30 days
Telem 2010	Retrospective	90	40 (44)	42	–	UC	Subtotal colectomy	0	61 (68)	End	11 (12)		30 days
Datta 2009	Retrospective	195	73 (37)	36	0	UC	Ileoanal pouch	133 (68)	–	Loop	86 (44)	9 (5)	30 days
Fowkes 2008	Prospective	32	14 (44)	42 (R 23–83)	–	UC	Subtotal colectomy	10 (31)	0	End	6 (19)	1 (3)	30 days
Kariv 2007	Case-matched	194	74 (38)	39	–	UC	IPAA	–	194 (100)	Loop	42 (22)	2 (1)	30 days
Schwenk 2006	Retrospective	29	16 (55)	65 (IQR 47–77)	11 (38)	Rectal cancer	LAR	29 (100)	10 (35)	Loop	7 (24)	2 (7)	30 days
Larson 2006	Case-matched	300	180 (60)	32 (R 17–66)	–	UC or FAP	IPAA	–	206 (69)	Loop	65 (22)	31 (10)	90 days
Garcia-Botello 2004	Prospective	127	54 (43)	54 (SD 19)	–	Colorectal disease	Any	–	–	Loop	2 (2)	1 (0.8)	Creation and closure
Hallbook 2002	Prospective	223	42 (19)	–	–	Colorectal disease	Any	223 (100)	–	Loop	11 (5)	3 (1)	Creation and closure
Okamoto 1995	Prospective	44	29 (65)	–	–	UC or FAP	IPAA	–	–	Both	–	3 (7)	Creation and closure
Wexner 1993	Prospective	83	31 (37)	45 (R 12–83)	–	Colorectal disease	Any	–	83 (100)	Loop	9 (11)	4 (5)	Creation and closure
Winslet 1991	Retrospective	34	18 (53)	33 (R 16–63)	–	Colitis/megacolon	IPAA	–	–	Loop	–	1 (3)	Creation and closure

RCT = randomized controlled trial; N = number; R = range; IQR = interquartile range; UC = ulcerative colitis; FAP = familial adenomatous polyposis, IPAA = ileal pouch-anal anastomosis; LAR = low anterior resection

### Readmission within 30 days

**Fig. 2:**
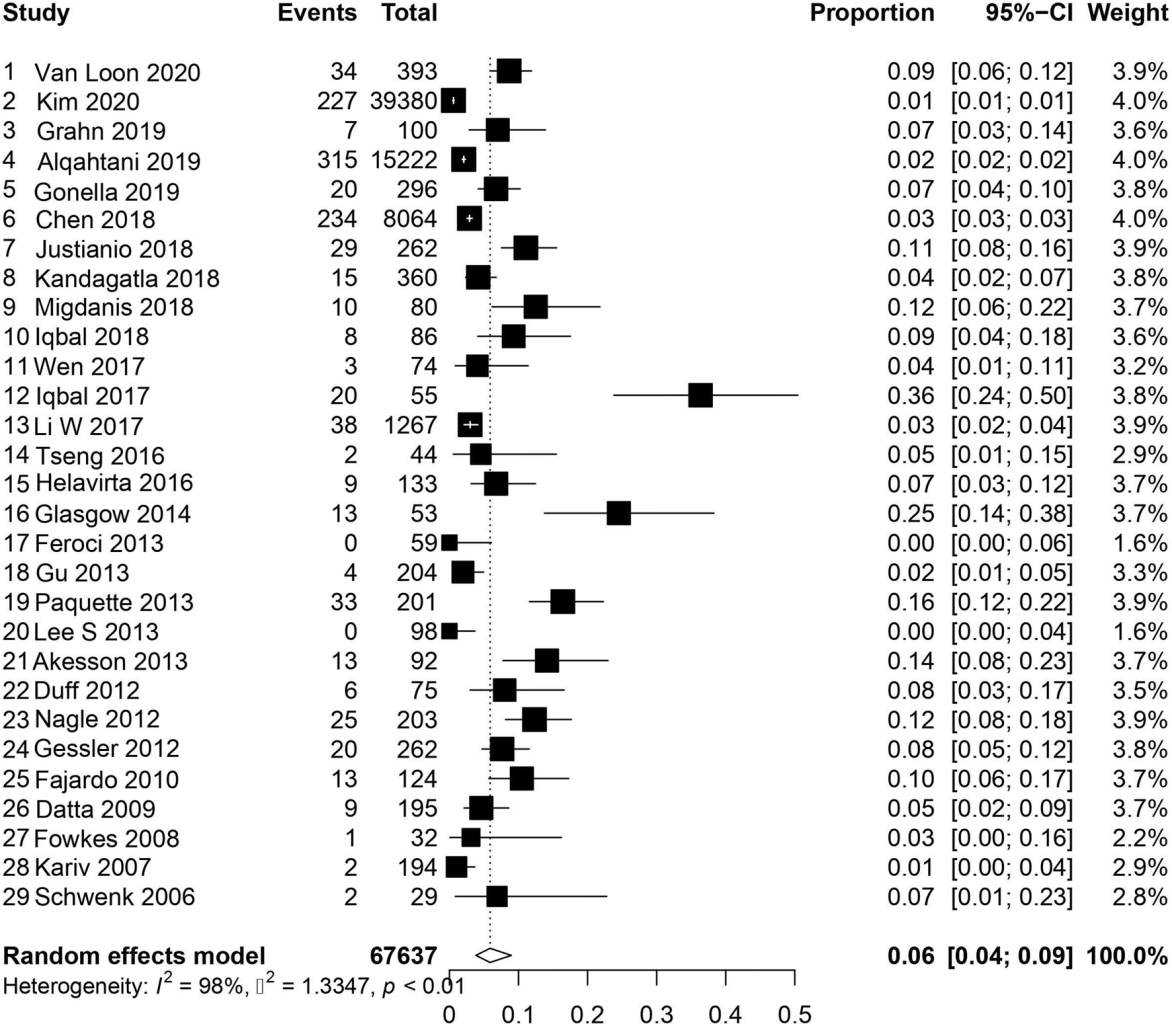
Readmission for dehydration within 30 days

**Fig. 3:**
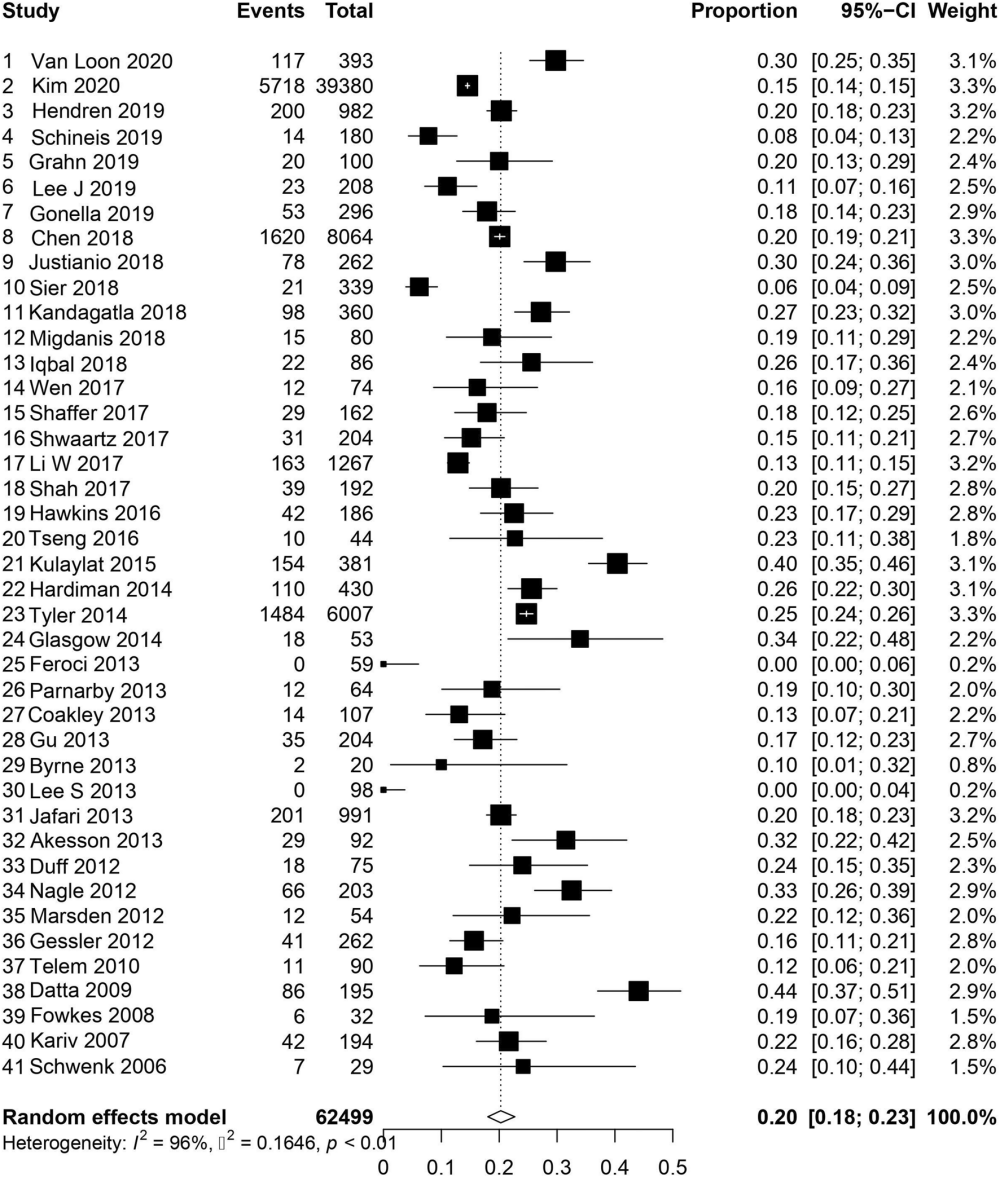
Overall readmission within 30 days

A total of 46 studies reported on readmission within 30 days of ileostomy creation [1, 2, 4–8, 11, 14–21, 23, 25, 26, 28–31, 33, 34, 36–38, 41–46, 52–55, 58, 60–66]. For those studies specifying readmission related to dehydration, the pooled incidence was 6% (95% CI 0.04–0.09, *I*^2^ = 98%, *τ*^2^ = 1.33 *p* < 0.01), Fig. 2 [1, 2, 6–8, 11, 16, 18–20, 23, 25, 26, 33, 37, 41, 42, 44–46, 54, 55, 58, 60, 61, 63–66]. For those studies reporting overall readmission rate, the pooled incidence was 20% (CI 95% 0.18–0.023, *I*^2^ = 96%, *τ*^2^ = 0.16 *p* < 0.01), Fig. 3 [1, 2, 4, 5, 7, 11, 14, 15, 17–21, 23, 25, 26, 28–31, 33, 34, 36–38, 41, 43–46, 52–55, 58, 60–64, 66]. For the studies assessing both overall and dehydration-related readmission, dehydration was the reason for readmission in 26% (95% CI 0.17–0.38, *I*^2^ = 97%, *τ*^2^ = 1.38 *p* < 0.01) of patients (Figure S1) [1, 2, 7, 11, 18–20, 23, 25, 26, 41, 44–46, 54, 55, 58, 60, 61, 63, 64, 66].

**Fig. 4:**
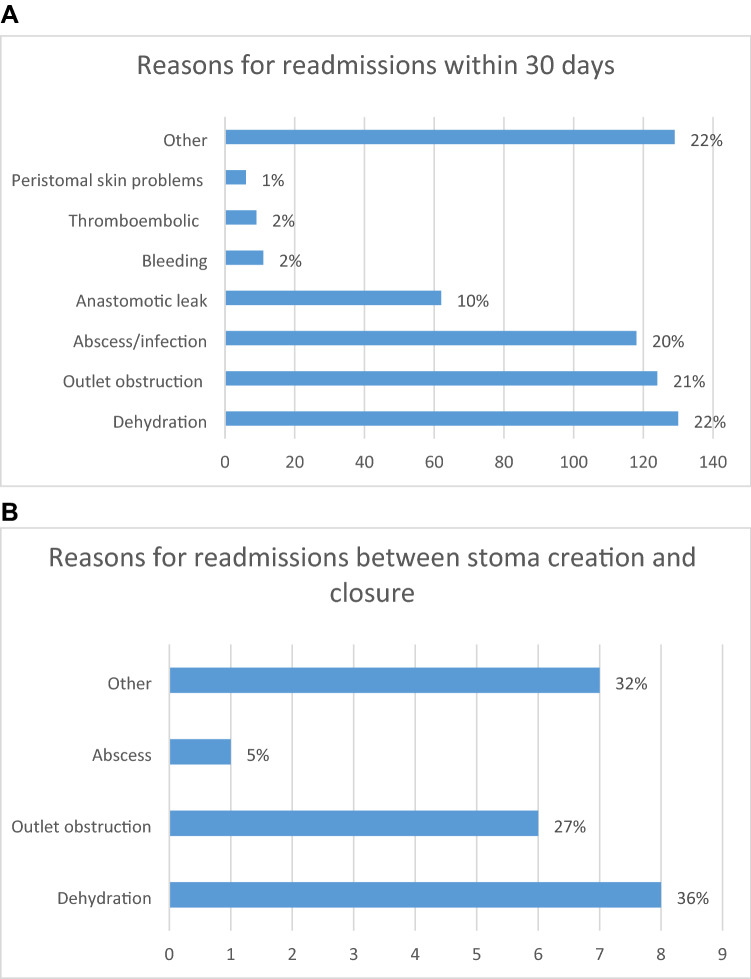
Reason for readmissions: **A** within 30 days. **B** Between stoma creation and closure

Other indications for readmission within 30 days were reported in 15 studies (Table 3 and Fig. 4) [1, 2, 11, 23, 25, 36, 44–46, 54, 55, 58, 61, 64, 66] and Kim et al. were removed from this section of the analysis, because more than half of the indications for readmission were unknown [2]. Dehydration was again the most common indication for readmission, with a pooled incidence of 5% (95%CI, 0.02–0.14, *I*^2^ = 98%, *τ*^2^ = 3.76 *p* < 0.01). Other indications for admission included stoma outlet issues in 4% (95% CI 0.02–0.08, *I*^2^ = 89%, *τ*^2^ = 0.98 *p* < 0.01) and infection (excluding anastomotic leaks) in 4% (95% CI 0.02–0.09, *I*^2^ = 96%, *τ*^2^ = 1.41 *p* < 0.01) (Figure S2).

**Table 3:** All reasons for readmission

Author	Number of readmissions	Dehydration *n*(%)	Outlet obstruction *n*(%)	Peristomal skin problems *n*(%)	Bleeding *n*(%)	Abscess/infection, *n*(%)	Thromboembolic *n*(%)	Anastomotic leak, *n*(%)	Other *n*(%)	Time frame
Hardt 2013	2	0	1 (1)					1 (1)		14 days
Van Loon 2020	117	34 (29)	26 (22)			35 (30)	6 (5)		16 (14)	30 days
Kim 2020	5718	227 (1)		170 (3)	4 (0.01)	914 (16)	212 (1)		4191 (73)	30 days
Grahn 2019	20	7 (7)				4 (4)			9 (45)	30 days
Kandagatla 2018	98	15 (4)				28 (8)			55 (56)	30 days
Iqbal 2018*	22	8 (9)	5 (6)			8 (9)			3 (14)	30 days
LI W 2017	163	38 (3)	42 (3)	1 (0.08)	4 (0.3)	42 (3)	3 (0.2)	14 (1)	19 (12)	30 days
Glasgow 2014	18	13 (25)	2 (4)						3 (17)	30 days
Gu 2013	35	4 (2)	12 (6)		2 (1)	1 (1)		12 (6)	4 (11)	30 days
Byrne 2013	2								2 (100)	30 days
Duff 2012	18	6 (8)	2 (3)	2 (3)				4 (5)	4 (22)	30 days
Nagle 2012*	66	25 (12)	19 (9)		2 (1)	19 (9)	2 (1)	3 (2)		30 days
Datta 2009	86	9 (5)	28 (14)					28 (14)	21 (24)	30 days
Fowkes 2008	6	1 (3)	2 (6)				1 (3)		2 (33)	30 days
Kariv 2007	42	2 (1)	12 (6)	2 (1)	2 (1)	14 (7)	3 (2)		7 (17)	30 days
Schwenk 2006	7	2 (7)		1 (3)	1 (3)	2 (7)		1 (3)		30 days
Charak 2018	36	14 (14)	2 (2)			12 (12)			8 (22)	60 days
Bednarski 2018	15	4 (8)	2 (4)			3 (6)		2 (4)	4 (27)	60 days
Fish 2017***	113	47 (12)	15 (4)			68 (17)			49 (43)	60 days
Phatak 2014	63	32 (11)	8 (3)	3 (1)			1 (0.3)	7 (2)	12 (19)	60 days
Messaris 2012	102	44 (7)	21 (4)		3 (1)	26 (4)	4 (1)		4 (4)	60 days
Park 2018	13	8 (11)	3 (4)						2 (15)	90 days
Larson 2006	65	31 (48)	6 (9)						28 (43)	90 days
Karjalainen 2019	50	19 (16)	9 (8)		1 (1)	6 (5)	1 (1)	2 (2)	12 (24)	3 months
Lee N 2020****	51	20 (39)	19 (37)			15 (29)			5 (10)	6 months
Anderin 2016	22	5 (4)	9 (7)					8 (6)		3 years
Garcia-Botello 2004	2	1 (1)							1 (1)	Creation and closure
Hallbook 2002	11	3 (1)	5 (2)						3 (27)	Creation and closure
Wexner 1993	9	4 (5)	1 (1)			1 (1)			3 (33)	Creation and closure

*Overlap in reason for readmission in two patients

**Overlap in reason for readmission in four patients

***Overlap in reason for readmission in 66 patients

****Overlap in reason for readmission in eight patients

### Readmission with 60 days

Readmission within 60 days of ileostomy creation was reported in 6 studies [3, 22, 32, 39, 49, 67]. Dehydration led to readmission in 10% (95% CI 0.08–0.12, *I*^2^ = 39%, *τ*^2^ = 0.02 *p* = 0.14), with the pooled proportion of all-cause readmission being 27% (95% CI 0.21–0.34, *I*^2^ = 88%, *τ*^2^ = 0.15 *p* < 0.01) (Figures S3, S4). Dehydration was the indication for readmission in 40% of all patients admitted during this timeframe (95% CI 0.34–0.47, *I*^2^ = 38%, *τ*^2^ = 0.04 *p* = 0.15), Figure S5.

Of the five papers reporting on other indications for readmission, Figure S6 [3, 22, 32, 39, 67], four mentioned dehydration as the leading cause [22, 32, 39, 67]. Other frequent indications included infection in 7% (95% CI 0.03–0.15, *I*^2^ = 92%, *τ*^2^ = 0.83 *p* < 0.01) and stoma outlet issues in 3% (95% CI 0.03–0.04, *I*^2^ = 0%, *τ*^2^ = 0 *p* = 0.89), Figure S7.

### Readmissions between stoma creation and closure

Eight studies reported on readmission related to dehydration between the time frame of ileostomy creation and closure (range 2–9 months) [27, 40, 47, 57, 68–70]. The pooled incidence of dehydration-related readmission during his time frame was 5% (95% CI 0.03–0.09, *I*^2^ = 65%, *τ*^2^ = 0.46 *p* < 0.01), Figure S8 [40, 47, 57, 68–70]. Five studies reported on all-cause readmissions, with an incidence of 11% (95% CI 0.04–0.26, *I*^2^ = 92%, *τ*^2^ = 1.25 *p* < 0.01), Figure S9 [27, 47, 57, 70]. Of all readmissions, dehydration was the indication in 37% (95% CI 0.19–0.59, *I*^2^ = 0%, *τ*^2^ = 0 *p* = 0.67), Figure S10 [47, 57, 70].

Of the 3 papers reporting specific indications for readmission during this time frame [47, 57, 70], 2% (95% CI 0.01–0.06, *I*^2^ = 53%, *τ*^2^ = 0.49 *p* = 0.12) were admitted for dehydration, 2% (95% CI 0.01–0.04, *I*^2^ = 0%, *τ*^2^ = 0 *p* = 0.45) for stoma outlet problems, and 1% (95% CI 0–0.02, *I*^2^ = 0%, *τ*^2^ = 0 *p* = 0.58) for infection (Figure S11).

### Duration of readmission

Ten studies reported on duration of readmission, as summarised in Table 4. Four studies reported specifically on admission for dehydration within 30 days with duration of readmission ranging from 2.5 to 6 days [6, 8, 11, 20]. Five studies reported on all-cause readmission, with duration ranging from 3 to 9 days [1, 11, 20, 25, 44]. In the remaining studies, duration of readmission within 60 days or between stoma creation and closure ranged from 5 to 9.5 days [3, 57, 67].

**Table 4:** Duration of readmissions

Study	Readmissions overall *N* (%)	Duration of readmission overall (days)	Readmissions dehydration *N* (%)	Duration readmission dehydration (days)	Time frame readmission (days)
Grahn 2019	20 (20)	4.7 (no range)	7 (7)	–	30 days
Justinianio 2018	78 (30)	6 (IQR 3–11)	29 (11)	6 (IQR 4–10)	30 days
Iqbal 2018	22 (26)	5 (IQR 13–31)	8 (9)	–	30 days
Fish 2017	113 (28)	5 (IQR 2–7)	47 (12)	4 (no range)	60 days
Iqbal 2017			20 (36)	4.2	30 days
Li W 2017	163 (13)	3 (rang 1–6)	38 (3)	4 (range 1–6)	30 days
Abegg 2014	32 (26)	9.5 (SD 6.6)	16 (14)	–	Creation and closure
Paquette 2013			33 (17)	2.4 (range 1–7)	30 days
Datta 2009	86 (44)	9.1 (no range)			30 days
Wexner 1993	9 (11)	5.2 (range 2–11)	4 (5)		Creation and closure

*IQR* interquartile range

### Cost of readmission for dehydration

Two studies reported readmission due to dehydration within 30 days of stoma creation, with a cost ranging between $2750 and $5924 per patient [6, 8]. If there was additional renal failure costs increased to $9107 [8]. After implementation of an ileostomy education and management protocol, one study reported a reduction in the number of readmissions specifically for dehydration from 65 to 16%, resulting in a mean costs saving of $63,821 ($25,037–$88,858) per year [6]. In the same hospital, the average cost of readmission for any cause was $13,839 per patient [25].

Shaffer et al. reported a total cost of $4,520 per patient for readmission within 30 days for any indication. After implementation of an intervention programme to improve monitoring, these costs were reduced to $508 per patient [5].

Tyler et al. reported a mean associated charge for readmission of $33,363 (SD, $89,396) for readmissions within 30 days after a colorectal resection. In patients with an ileostomy, acute renal failure and fluid and electrolyte disorders were the second most common cause of readmission (17.4%) after surgical complications directly related to the procedure (19.3%) [4].

## Discussion

In the present systematic review and meta-analysis, the readmission rate within 30 days after stoma creation is 20%, with dehydration as the leading cause, occurring in around 6% of patients [1–3, 11, 22, 32, 39, 56, 58, 61]. Other frequent indications for readmission include stoma outlet issues and infection, both occurring in around 4% of patients. The average cost of readmission is high with dehydration-related readmission costing between $2750 and $5,924 per patient. Thus, the creation of an ileostomy is associated with a risk of complications that frequently require costly readmission.

This high readmission rate following the creation of an ileostomy is consistent with previous published data. However, data examining the factors associated with readmission are still limited to small cohorts, single institutions, or are from reports often of poor quality [1, 2, 11]. Nonetheless dehydration, stoma outlet obstruction, and infection have been cited repeatedly as the most frequent causes.

Dehydration is most common in the early post-operative period, with the highest incidence of reduced kidney function within the first 3–6 months after surgery [48, 63, 68, 69]. Some authors report that estimated glomerular filtration rate (eGFR) values post-closure closely resemble the normal preoperative situation [69]. Others have shown a significant reduction in eGFR after ileostomy creation which remains present up to 12 months after ileostomy closure [48, 70]. Fielding et al. found that a decline in kidney function after ileostomy creation resulted in an increased risk of severe chronic kidney disease [CKD] ≥ 3, OR 6.89 (95% CI 4.44–10.8, *p* < 0.0001) [48]. Dehydration after creation of an ileostomy may therefore have a significant impact on patient morbidity.

Risk factors for dehydration include: stoma output more than 1 L at discharge [20], the presence of comorbidity [16, 18], a higher American Society of Anesthesiologists (ASA) classification [2, 19, 23], older age [8, 19, 20], smoking [16], hypertension [19], diabetes [2, 16], use of diuretics [20, 22, 39], and chemotherapy [11, 20]. The influence of gender is unclear. One study reported that female gender was associated with an increased risk for readmission for dehydration (OR 1.59) [19], and another report showed that men were more likely to be readmitted for this reason (OR 3.18) [20]. Some consider enhanced recovery after surgery (ERAS) may lead to a higher rate of readmission, but from the limited evidence available, this has not been confirmed [33, 35–37, 46, 55]. In any case, such programmes should focus on minimizing post-operative complications, preparing patients for discharge, and arranging adequate outpatient support.

Readmissions are costly and may be avoidable to some extent. This is particularly the case for dehydration, since better monitoring and timely intervention might prevent extensive fluid loss. Improved inpatient coaching and outpatient follow-up care have been shown to reduce readmission [1, 6, 18, 30, 64]. Despite attempts by others to introduce such programmes readmission rates remain high in some of the studies [6, 66]. Many of these studies had very small sample sizes [1, 6], and the reduction of readmissions after implementation of the protocol did not always reach a statistically significant level [1, 30]. Therefore, from these data, post-operative care pathways may offer a solution to the problem, but there is a need for further high-quality research to standardize the approach.

There are some limitations to this review. In most studies, readmission rates were not the primary outcome of the study. This might have led to under-reporting. There was significant heterogeneity between the different studies, making the results prone to information bias. This heterogeneity can partly be attributed to the variety of ileostomy indications in different patient populations, and the time span of 30 years in this systematic review which might include changes in indication and management of an ileostomy. In addition, the definition of dehydration and the method of diagnosis varied; for example in some studies, coded diagnoses were used to identify patients with dehydration. In this review, the majority of the ileostomies were created in an elective setting [7, 21, 22, 24–26, 29, 32, 33, 35, 36, 38, 40, 42, 46–48, 59]. This might have led to an underestimate readmission as emergency surgery is known to increase complications. Furthermore, there were only a few reports on preoperative kidney function, or other factors that might contribute to the risk of dehydration such as an additional small bowel resection or post-operative re-intervention. Finally, the reason for readmission within 30 days was unknown in 62% of the largest cohort included in our meta-analysis [2].

## Conclusions

One out of five patients is readmitted after creation of an ileostomy. Dehydration is the leading cause for these readmissions, occurring in one-third of patients within 30 days. This comes with high health care costs. Better monitoring, patient awareness, and preventive measures are required.

## Supplementary Information

Below is the link to the electronic supplementary material.

Supplementary file1 (DOCX 13212 KB) Supplementary Figure 1: Proportion of readmission for dehydration of overall readmissions within 30 days. Supplementary Figure 2: Most common causes of readmission within 30 days A. dehydration B Stoma outlet problems C. Infection. Supplementary Figure 3: Readmissions for dehydration within 60 days. Supplementary Figure 4: Overall readmissions within 60 days. Supplementary Figure 5: Proportion of readmission related to dehydration of overall readmissions. Supplementary Figure 6: All causes readmission dehydration within 60 days. Supplementary Figure 7: All causes readmission dehydration within 60 days. Supplementary Figure 8: Readmissions related to dehydration between stoma creation and closure. Supplementary Figure 9: Overall readmissions between stoma creation and closure. Supplementary Figure 10: Proportion of readmission for dehydration of overall readmissions. Supplementary Figure 11: Most common causes of readmission between stoma creation and closure A. dehydration B. Stoma outlet problems C. Stoma infection.
