# Supplementary material for: Overall readmissions and readmissions related to dehydration after creation of an ileostomy: a systematic review and meta-analysis
Source: Tech Coloproctol. 2022 Feb 22;26(5):333–49. doi: 10.1007/s10151-022-02580-6 (PMC9018644; doi:10.1007/s10151-022-02580-6)
Supplement: Supplementary file 1 — Supplementary file1 (DOCX 13212 KB) Supplementary Figure 1: Proportion of readmission for dehydration of overall readmissions within 30 days. Supplementary Figure 2: Most common causes of readmission within 30 days A. dehydration B Stoma outlet problems C. Infection. Supplementary Figure 3: Readmissions for dehydration within 60 days. Supplementary Figure 4: Overall readmissions within 60 days. Supplementary Figure 5: Proportion of readmission related to dehydration of overall readmissions. Supplementary Figure 6: All causes readmission dehydration within 60 days. Supplementary Figure 7: All causes readmission dehydration within 60 days. Supplementary Figure 8: Readmissions related to dehydration between stoma creation and closure. Supplementary Figure 9: Overall readmissions between stoma creation and closure. Supplementary Figure 10: Proportion of readmission for dehydration of overall readmissions. Supplementary Figure 11: Most common causes of readmission between stoma creation and closure A. dehydration B. Stoma outlet problems C. Stoma infection. [file 10151_2022_2580_MOESM1_ESM.docx]

**Appendix**

Date of search: 24-04-2020

Search updated: 19-4-2020

**Supplementary Table 1**

Database(s): **Medline (via OVID)** 1990-present

| **#** | **Searches** |
| --- | --- |
| 1 | (Dehydration OR high output OR complication OR acute kidney injury OR kidney function OR renal function OR renal dysfunction OR renal failure OR kidney dysfunction OR acute renal injury OR kidney failure). ti,ab,kw. |
| 2 | (Readmission) ti,ab,kw. |
| 3 | (Ileostomy OR Surgical stoma OR Diverting stoma) ti,ab,kw. |
|  | 1 or 2 and 3 |
| **Total hits** | **3414** |

**Supplementary Table 2**

Database(s): **Embase Classic+Embase (via OVID)** 1990-present

| **#** | **Searches** |
| --- | --- |
| 1 | (Dehydration OR high output OR complication OR acute kidney injury OR kidney function OR renal function OR renal dysfunction OR renal failure OR kidney dysfunction OR acute renal injury OR kidney failure).ti,ab,kw. |
| 2 | (Readmission) ti,ab,kw. |
| 3 | (Ileostomy OR Surgical stoma OR Diverting stoma) ti,ab,kw. |
|  | (1 OR 2) AND 3 |
| **Total hits** | **2250** |

**Supplementary Table 3**

Database(s): **Cochrane Library** 1990-present

| **#** | **Searches** |
| --- | --- |
| 1 | Mesh descriptor: [Readmissions] explode all trees |
| 2 | Mesh descriptor: [Dehydration] explode all trees |
| 3 | Mesh descriptor: [Acute Kidney Injury] explode all trees |
| 4 | (Dehydration OR high output OR complication OR acute kidney injury OR kidney function OR renal function OR renal dysfunction OR renal failure OR kidney dysfunction OR acute renal injury OR kidney failure). ti,ab,kw. (word variations have been searched) |
| 5 | 1 OR 2 OR 3 OR 4 |
| 6 | Mesh descriptor: [Ileostomy] explode all trees |
| 7 | (ileostomy OR surgical stoma OR diverting stoma) ti,ab,kw. (word variations have been searched) |
| 8 | 6 OR 7 |
| 9 | 5 AND 8 |
| **Total hits** | 15 hits (8 Cochrane reviews, 2 Protocols, 5 Trials) |

**Supplementary figure 1 Proportion of readmission for dehydration of overall readmissions within 30 days**


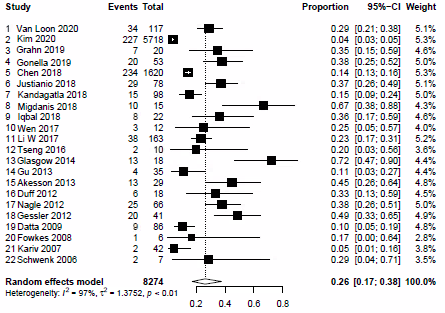


**Supplementary figure 2 Most common causes of readmission within 30 days A. dehydration B Stoma outlet problems C. Infection**

**A.**


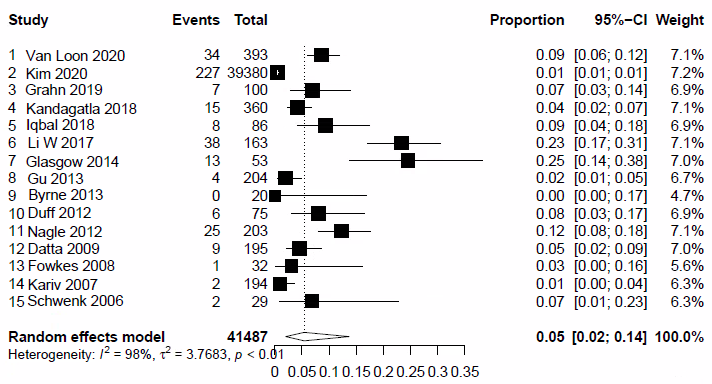


**B.**


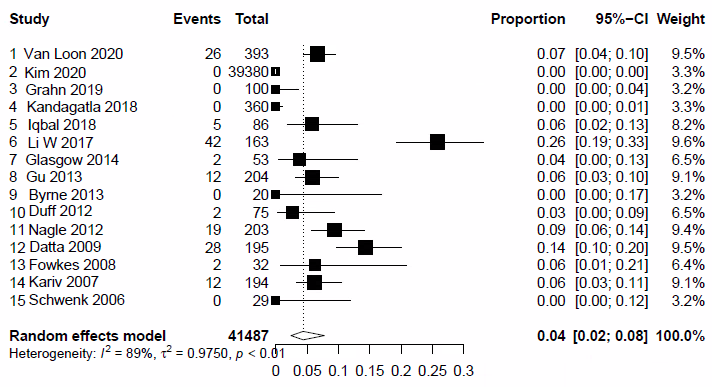


**C.**
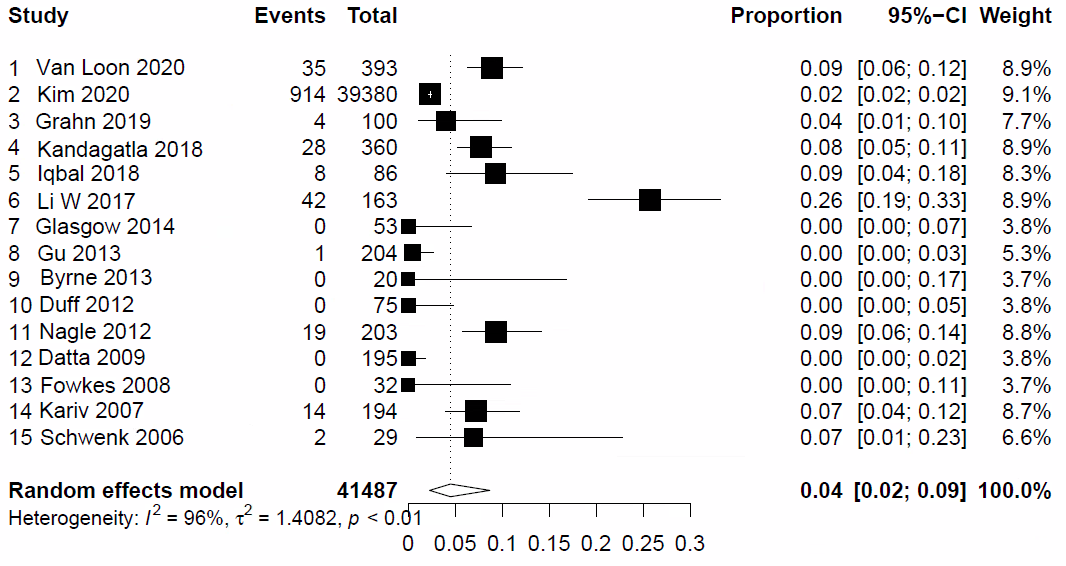


**Supplementary figure 3 Readmissions for dehydration within 60 days**


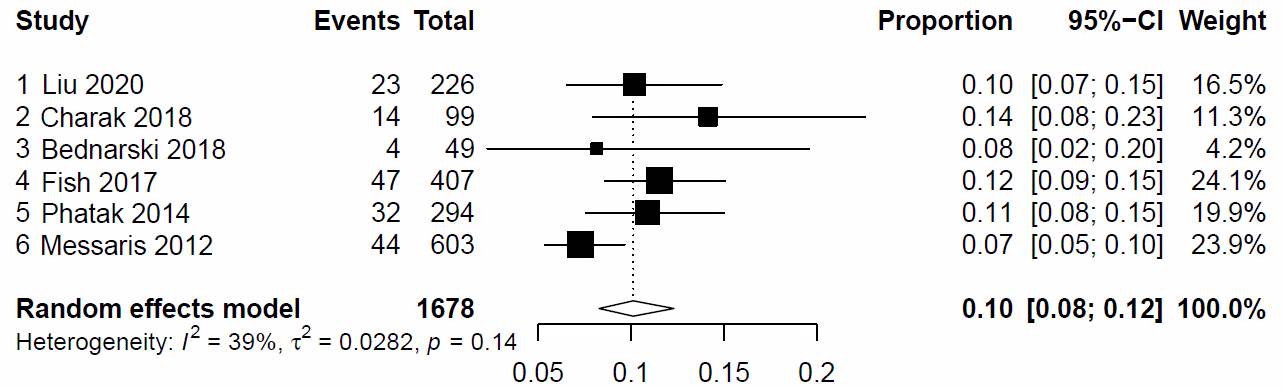


**Supplementary figure 4 Overall readmissions within 60 days**


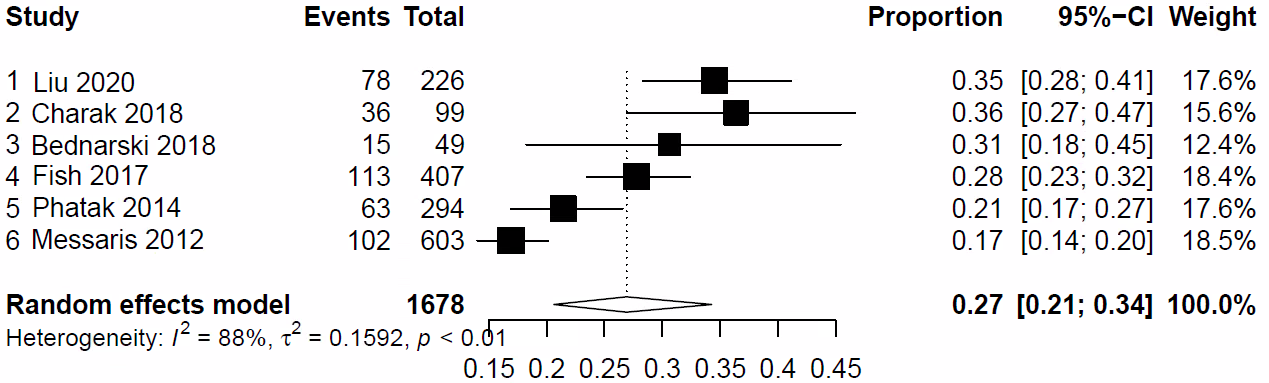


**Supplementary figure 5 Proportion of readmission related to dehydration of overall readmissions**


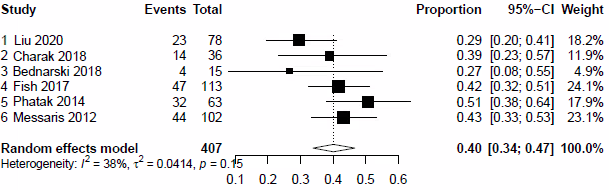


**Supplementary figure 6 All causes readmission dehydration within 60 days**

**Supplementary figure 7 Most common causes for readmission within 60 days A. dehydration B. Stoma infection C. Stoma outlet problems**

**A.**


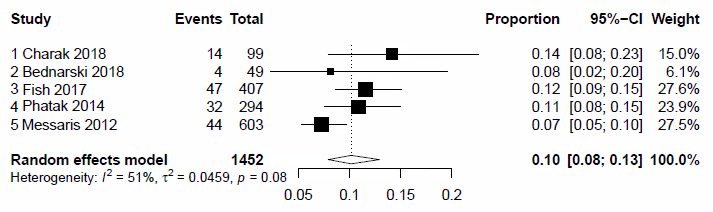


**B.**

**
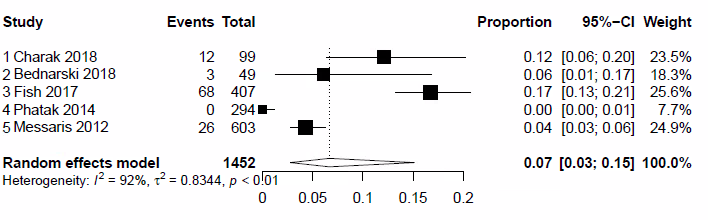
**

**C.**


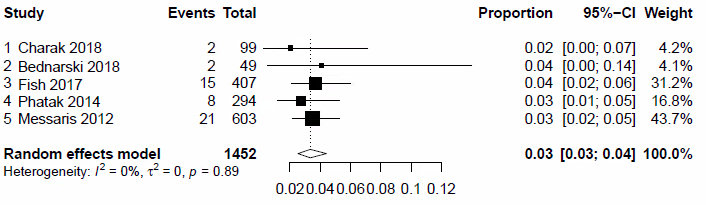


**Supplementary figure 8 Readmissions related to dehydration between stoma creation and closure**


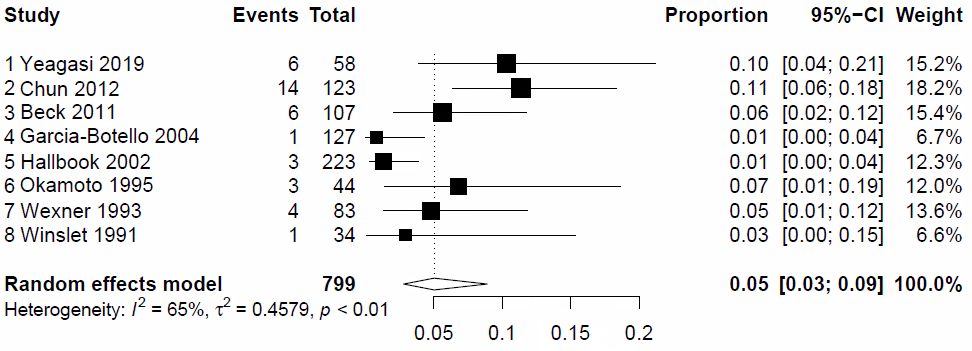


**Supplementary figure 9 Overall readmissions between stoma creation and closure**


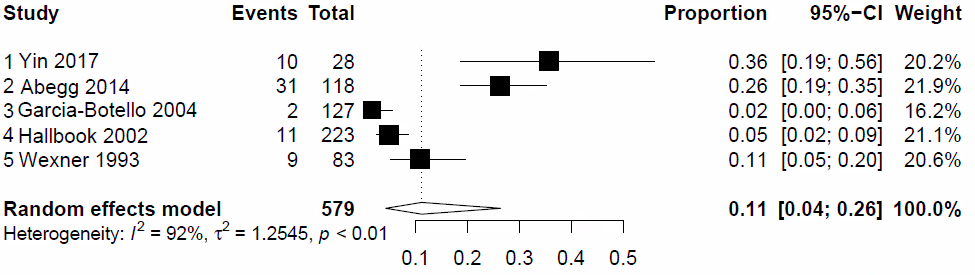


**Supplementary figure 10 Proportion of readmission for dehydration of overall readmissions**


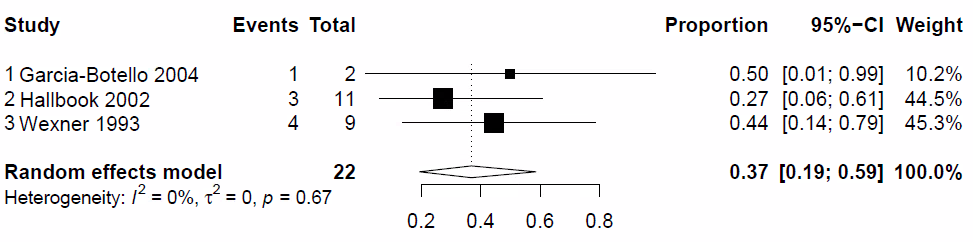


**Supplementary figure 11 Most common causes of readmission between stoma creation and closure A. dehydration B. Stoma outlet problems C. Stoma infection**

**A.**


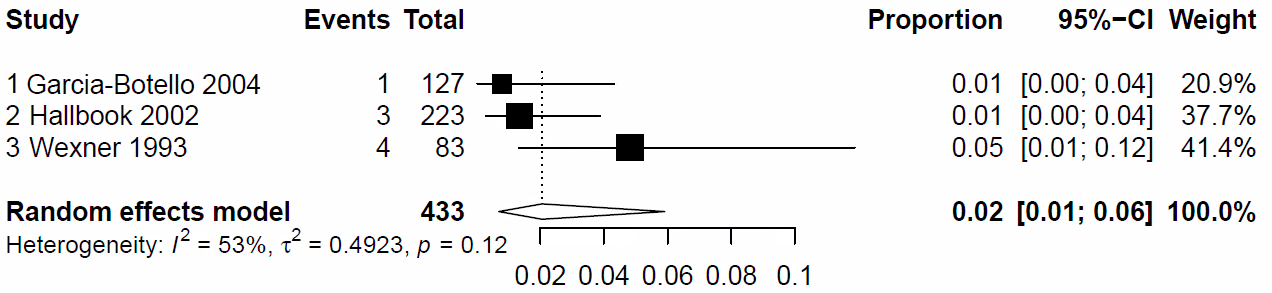


**B.**


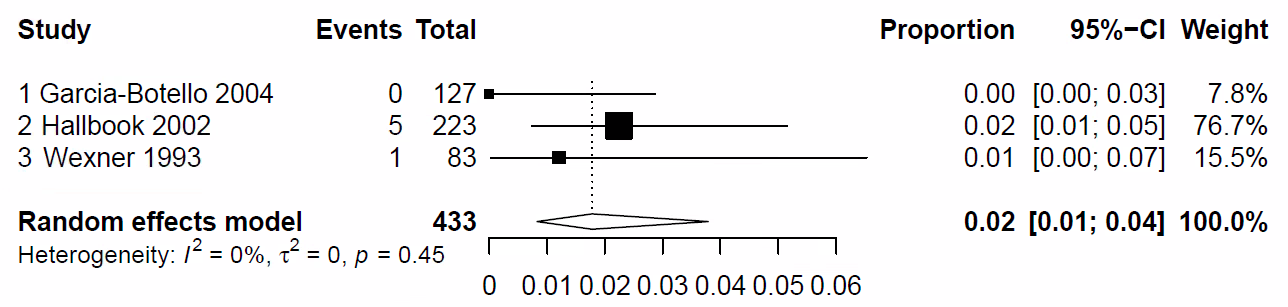


**C.**


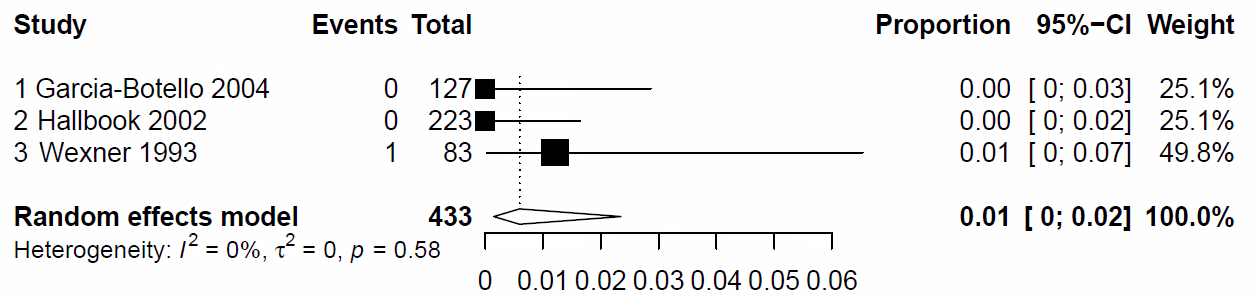


**MOOSE Checklist for Meta-analyses of Observational Studies**

| **Item No** | **Recommendation** | **Reported on Page No** |
| --- | --- | --- |
| Reporting of background should include | | |
| 1 | Problem definition | 3 |
| 2 | Hypothesis statement | 3 |
| 3 | Description of study outcome(s) | 3 |
| 4 | Type of exposure or intervention used | 5 |
| 5 | Type of study designs used | 4 |
| 6 | Study population | 4 |
| Reporting of search strategy should include | | |
| 7 | Qualifications of searchers (eg, librarians and investigators) | 1 |
| 8 | Search strategy, including time period included in the synthesis and key words | 4 |
| 9 | Effort to include all available studies, including contact with authors | 4 |
| 10 | Databases and registries searched | 4 |
| 11 | Search software used, name and version, including special features used (eg, explosion) | 4 |
| 12 | Use of hand searching (eg, reference lists of obtained articles) | 4 |
| 13 | List of citations located and those excluded, including justification | 6 + figure 1 |
| 14 | Method of addressing articles published in languages other than English | 4 |
| 15 | Method of handling abstracts and unpublished studies | 4 |
| 16 | Description of any contact with authors | - |
| Reporting of methods should include | | |
| 17 | Description of relevance or appropriateness of studies assembled for assessing the hypothesis to be tested | 4 |
| 18 | Rationale for the selection and coding of data (eg, sound clinical principles or convenience) | 4-5 |
| 19 | Documentation of how data were classified and coded (eg, multiple raters, blinding and interrater reliability) | 4-5 |
| 20 | Assessment of confounding (eg, comparability of cases and controls in studies where appropriate) | 4-5 |
| 21 | Assessment of study quality, including blinding of quality assessors, stratification or regression on possible predictors of study results | 6 |
| 22 | Assessment of heterogeneity | 6 |
| 23 | Description of statistical methods (eg, complete description of fixed or random effects models, justification of whether the chosen models account for predictors of study results, dose-response models, or cumulative meta-analysis) in sufficient detail to be replicated | 6 |
| 24 | Provision of appropriate tables and graphics |  |
| Reporting of results should include | | |
| 25 | Graphic summarizing individual study estimates and overall estimate | Appendix |
| 26 | Table giving descriptive information for each study included | S5 |
| 27 | Results of sensitivity testing (eg, subgroup analysis) | 7-9 |
| 28 | Indication of statistical uncertainty of findings | 7-9 |

| **Item No** | **Recommendation** | **Reported on Page No** |
| --- | --- | --- |
| Reporting of discussion should include | | |
| 29 | Quantitative assessment of bias (eg, publication bias) | 7-9 |
| 30 | Justification for exclusion (eg, exclusion of non-English language citations) | Figure 1 |
| 31 | Assessment of quality of included studies | 6 + Appendix table 4 |
| Reporting of conclusions should include | | |
| 32 | Consideration of alternative explanations for observed results | 11-12 |
| 33 | Generalization of the conclusions (ie, appropriate for the data presented and within the domain of the literature review) | 13 |
| 34 | Guidelines for future research | 12-13 |
| 35 | Disclosure of funding source | 1 |

*From*: Stroup DF, Berlin JA, Morton SC, et al, for the Meta-analysis Of Observational Studies in Epidemiology (MOOSE) Group. Meta-analysis of Observational Studies in Epidemiology. A Proposal for Reporting. *JAMA*. 2000;283(15):2008-2012. doi: 10.1001/jama.283.15.2008.
